# Engineered peptide-based nanobiomaterials for electrochemical cell chip

**DOI:** 10.1186/s40580-016-0077-7

**Published:** 2016-07-25

**Authors:** Md. Abdul Kafi, Hyeon-Yeol Cho, Jeong-Woo Choi

**Affiliations:** 1 Department of Microbiology and Hygiene, Bangladesh Agricultural University, Mymensigh, 2202 Bangladesh; 2 Interdisciplinary Program of Integrated Biotechnology, Sogang University, Seoul, 04107 South Korea; 3 Department of Chemical and Biomolecular Engineering, Sogang University, Seoul, 04107 South Korea

**Keywords:** Engineered peptide, RGD peptide, Nanobiomaterials, Cell chip, Electrochemical monitoring

## Abstract

Biomaterials having cell adhesion ability are considered to be integral part of a cell chip. A number of researches have been carried out to search for a suitable material for effective immobilization of cell on substrate. Engineered ECM materials or their components like collagen, Poly-l-Lysine (PLL), Arg-Gly-Asp (RGD) peptide have been extensively used for mammalian cell adhesion and proliferation with the aim of tissue regeneration or cell based sensing application. This review focuses on the various approaches for two- and three-dimensionally patterned nanostructures of a short peptide i.e. RGD peptide on chip surfaces together with their effects on cell behaviors and electrochemical measurements. Most of the study concluded with positive remarks on the well-oriented engineered RGD peptide over their homogenous thin film. The engineered RGD peptide not only influences cell adhesion, spreading and proliferation but also their periodic nano-arrays directly influence electrochemical measurements of the chips. The electrochemical signals found to be enhanced when RGD peptides were used in well-defined two-dimensional nano-arrays. The topographic alteration of three-dimensional structure of engineered RGD peptide was reported to be suitably contacted with the integrin receptors of cellular membrane which results indicated the enhanced cell-electrode adhesion and efficient electron exchange phenomenon. This enhanced electrochemical signal increases the sensitivity of the chip against the target analytes. Therefore, development of engineered cellular recognizable peptides and its 3D topological design for fabrication of cell chip will provide the synergetic effect on bio-affinity, sensitivity and accuracy for the in situ real-time monitoring of analytes.

## Introduction

Recently, cell chip based electrochemical sensing has been proved to be a potential tool for bio sensing [1], environmental monitoring [2–4], and in vitro drug effect studies [5, 6]. This label free detection method provides accurate, in situ monitoring of analytes avoiding photo bleaching effect of the traditional colorimetric spectrochemical assays [7]. This important tool requires mammalian cell immobilized platform on which the analytes were exposed prior to electrochemical recoding of the cellular responses. The treatment of analytes and recording cellular response required several washing steps that might cause cell eruption from the electrode surface. To avoid such possibility, farm cell-electrode attachment should be ensure for effective sensing of analytes. Therefore, bioengineers are still looking for suitable material with superior cell adhesion ability. Hence, this review focuses on the applications of engineered cell adhesion molecules at the cell electrode interfaces with special emphasis on the effects of RGD motif at various two- and three-dimensionally patterned nanostructures on the sensitivity of electrochemical measurements.

Materials of biological origin i.e. biomaterials have numerous applications in vitro biological or biomedical or tissue engineering research. In the recent decades, a number of researches have been carried out on the use of biomaterials with in vivo like functionality for tissue engineering applications [8–10]. The roles of biomaterials found to be depends on their two or three dimensional topography as well as their nano or microscale spatial arrangements [11–13]. The nanoscale arrangements with desired topography of biomaterials proved to be potential for specific function in advancing the field of biology and medicine. Like in vivo condition, the precise arrangement of nanostructured extra cellular matrix (ECM) materials allows their adhesion motif to the cellular receptors and found to posse significant influences on cell functions [12–14]. Hence, establishment of nanostructured biomaterials on the artificial surface is pivotal for the fabrication of living cell based bioplatform for tissue engineering and sensing application. The repeated unites of Arg-Gly-Asp (RGD) in ECM proteins are considered to be functional motif for cell-ECM interaction through RGD integrin linkage [15–17]. Recently, this RGD tripeptide sequences were synthesized at a various archistructural arrangements like RGD-Map-C and C(RGD)_4_ for the functionalization of artificial surface for establishing mammalian cells [10, 18]. The RGD motif organized on the artificial surfaces at a define nanoscale arrangement is essential for the sensing applications of fabricated cell based platform.

The nanoscale patterning of biomaterials has become an important topic in cell chip based research. Recently, RGD tripeptide sequences are patterned at various spatial or archistructural arrangements using the self-assembling (commonly known SA method) of biomaterials with several copolymers or guided assembling of the materials through size controlled porous masks (mask guided self-assembly, MGSA method) [10, 11]. The former SA method allows the definite spatial arrangement by controlling the ratio between the materials and copolymers [10]. However, recently introduced modified self-assembly method i.e. MGSA has given an excellent opportunity for assembling biomaterials at a definite spatial as well as archistructural arrangements [11]. This precise spatial and size controlled nanostructuring opportunity together with the excellent cell adhesion ability of RGD peptide has great significance in the rapidly expanding cell chip technologies [19].

Therefore, this review discusses on the use of several biomaterials at cell electrode interfaces of a living cell based chip together with their influence on cellular adhesion as well as on the electrochemical measurements. In addition, the detailed method of establishment of cell adhesion molecules with special emphasis on RGD nano-structuring protocol has been discussed here in this review. The performances of various materials modified surfaces have been discussed with special emphasis on their cellular adhesion and electrochemical measurements. Moreover, the effect of RGD nanostructures and their homogenous thin film like arrangement has been discussed here critically to suggest a suitable RGD nanostructure for cell chip. This article recommends that cell immobilized on RGD-Map-C nanostructures modified conductive cell chip is very effective tool for the electrochemical measurement.

## Biomaterials used at cell electrode interface for cell chip fabrication

Cell in tissue produces extracellular materials on which it is strictly attached, proliferates and organized to attain a specific tissue structure. ECM materials or their component like collagen, PLL, RGD peptides have been extensively used for engineering of artificial bioplatform suitable for mammalian cell adhesion and proliferation with the aim of cell based sensing applications or tissue regenerations [20–23].

### Collagen

**Fig. 1:**
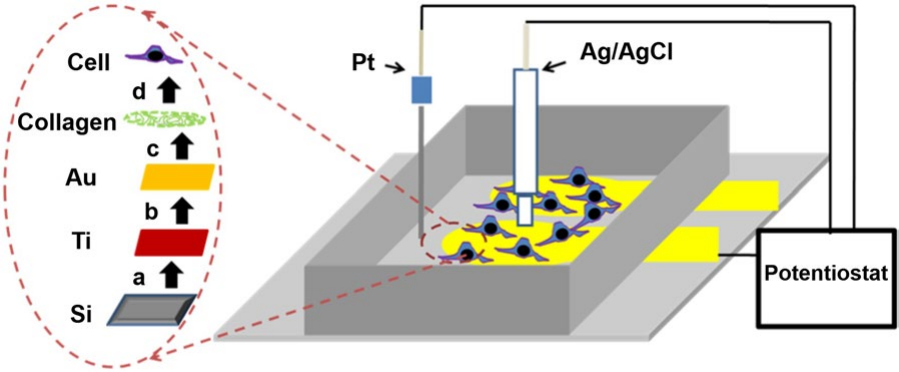
Schematics of a cell-chip: the *dotted circle* shows the steps of fabrication,* a* sputtering of 15 nm titanium on silicon,* b* establishment of 150 nm Au,* c* collagen coating and* d* cell seeding. Reprinted with permission from: Ref. [28]. Copyright@American Scientific Publishers

Collagen is a fibrillar protein occurs as major functional component of ECM and considered as the most abundant protein in mammals. Naturally collagen occurs as long fibers of triple stranded helical structure with a length of 300 nm and thickness of 1.5 nm [24]. Three of the so called α-chains are twisted together to form the long rope-like helix. So far known 20 different types of collagen are formed by the triple helix of 25 different types of α- chain [25, 26]. The actual arrangement of the collagen in tissues is influenced by the cell embedded within, which scroll over it and pull on the ECM components. Among this numerous types collagen type-1, type-II and type-IV are most commonly used for three dimensional networking [26, 27]. The most remarkable property of collagen is their resistance toward strong tensile forces [25]. Therefore, these collagen types are commonly using in three artificial surfaces for the enhancement of the cell adhesion ability as shown in Fig. 1 [28]. However, their application to cell chip faces challenges due to their long fibrous structure [24]. The minimum thickness of collagen layer is 300 nm theoretically [24] and it easily forms the aggregated structure on chip surface. For this reason, the collagen layer can works as an insulator on the electrode surface and results the decreased sensitivity of cell chip. Therefore, researchers were searching for suitable alternative to the collagen for effective to bind with integrin without hampering electron exchange mechanism.

### PLL peptide

**Fig. 2:**
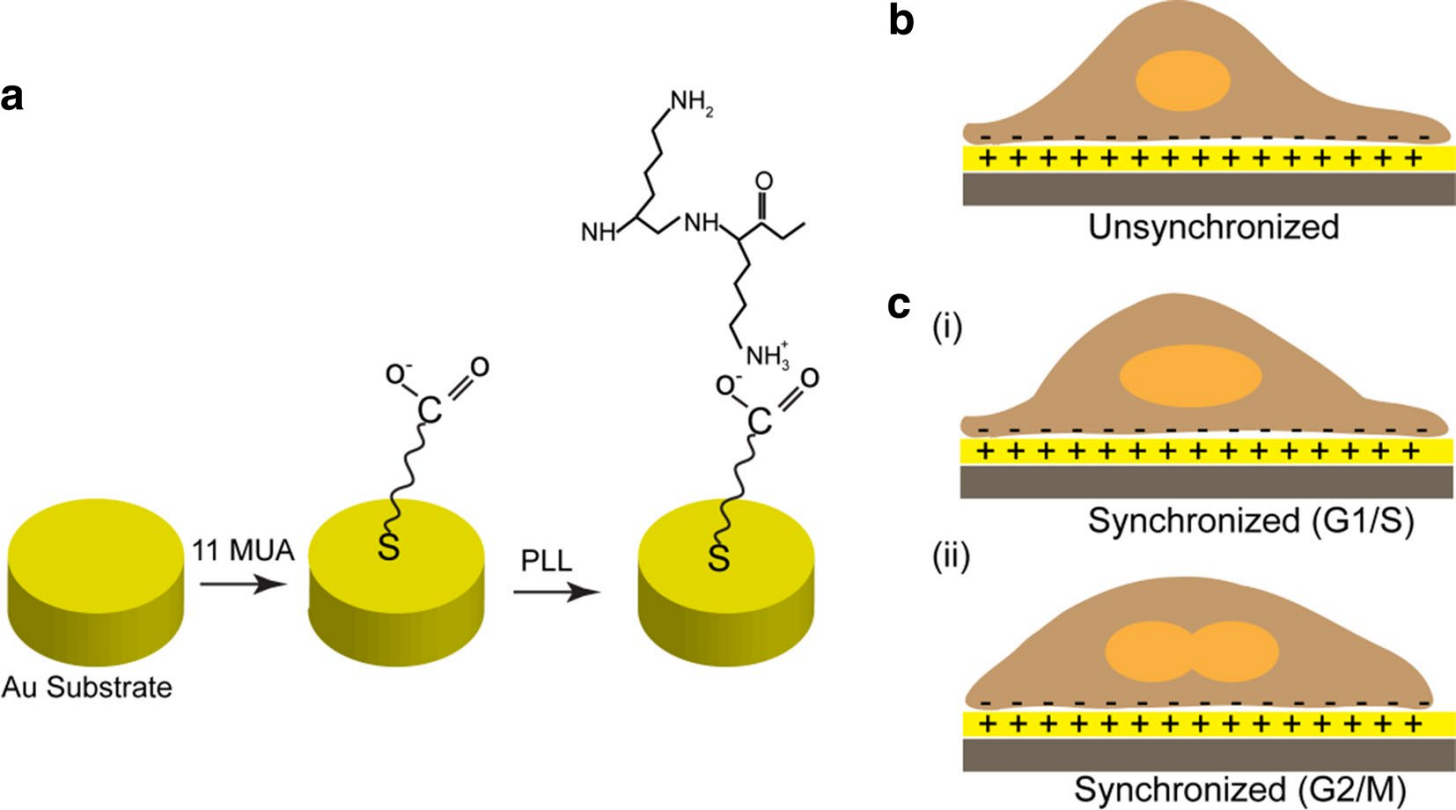
Immobilization of PLL on 11-MUA functionalized Au surface. **a** PLL conjugation on MUA self-assembled Au surface. Electro-statically immobilized **b** unsynchronized cell and **c** cell synchronized at G1/S *(i)* and G2/M phase *(ii)*. Figure reproduced with permission from: Ref. [4], Copyright (2013) with permission from Elsevier

As an alternative of whole ECM or collagen, some non-native proteins or peptides like PLL were used for the immobilization live cell on the metal-electrode surfaces for the suitability of electron exchange phenomena between cell-electrode. It is reported that lysine sequences of PLL polypeptide involve in the cell adhesion mechanism and enhances neuronal adhesion, proliferation, differentiation and neurite extension [29]. PLL peptide do not mediate receptor mediated adhesion rather it modulates cell adhesion via non receptor mediated electrostatic binding mechanism [4]. The electrode surface functionalized with PLL showed positive charge due to positive charged lysine. On the other hand, it is well known that naturally cell possess negative charge at their surfaces. Therefore, positively charged PLL peptide modified electrode attracts negatively charged cell membrane results farm electrostatic interaction. The cell membrane possesses negative charge due to the presence of glyco-calyx which encompasses short oligosaccharide chains containing a large number of sialic acid residues [30]. Recently, PLL peptide immobilized gold (Au) surface was used for the immobilization of neural cell for cell chip based sensing application [4]. Where, Au surface was pre-functionalized with 11-mercaptoundecanoic acid (11-MUA) self-assembled monolayer for the effective immobilization of PLL molecules (Fig. 2).

### RGD peptide

**Fig. 3:**
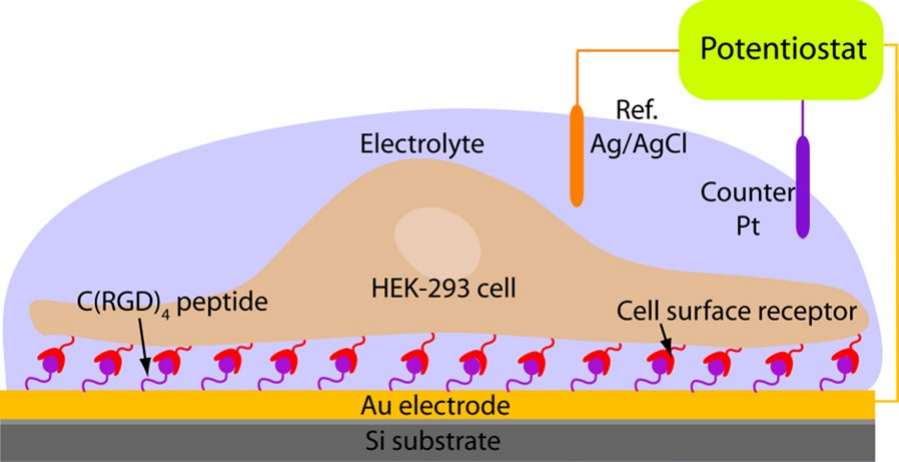
Receptor mediated cell immobilization, where RGD integrin coupling in cell adhesion process. Figure reproduced with permission from: Ref. [3], Copyright (2010) with permission from Springer

ECM provides three dimensional archistructural for cell through adhesion molecules (AMs) including laminin, collagen and initiate receptor mediated cell binding to attain three dimensional tissue structures [31]. The adhesion molecule in ECM is composed of RGD tripeptide enriched sequences which involve in covalent linkage with the αV-ß_3_ integrin receptors of cell surfaces [15]. The focal adhesion molecules are not only involved in the cell adhesion process but also in the two-way signal transfer (i.e. into and out of the cell) through an elaborate mechanotransduction system as shown in Fig. 3 [32]. Considering the integrin specificity and suitability in signal transfer, the short RGD tripeptide sequence has been attracted attention of the bioengineer for establishing living cell on the artificial surface. Recently, a series of researches have been performed with the RGD peptide based functionalization of metal electrode surface to establish living cells for their electrochemical sensing applications [4, 10, 11, 18]. Given that isolation of small RGD sequences from the large ECM proteins is a complex process, the short RGD peptide sequence was synthesized in the laboratory using the recombinant genetic engineering [10]. The synthetic RGD peptide sequences have been proved successful for the establishment of strong link at the metal-cell interface [10]. Moreover, the synthetic peptide showed better stability with less steric hindrance of signal transmission than the whole proteins [12].

**Fig. 4:**
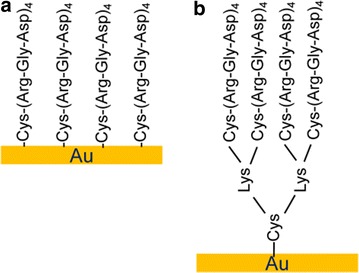
Schematics of the C(RGD)_4_ (**a**) and RGD-MAP-C (**b**) peptide immobilized on Au surface. Figure reproduced with permission from: Ref. [45], Copyright (2007) with permission from The Korean BioChip Society (2007)

Considering the suitable potential, RGD tripeptide sequence was further designed into several polymorphs (C(RGD)_4_ and RGD-MAP-C) and were synthesized from Peptron (Korea) as shown in Fig. 4 [10]. Where, peptides were prepared by solid phase peptide synthesis using standard 9-fluorenylmethyloxyccarbonyl (Fomc) chemistry. High-performance liquid chromatography (HPLC) analysis indicated that the synthetic peptides were at least 95 % pure. The peptides were dissolved in phosphate buffered saline (PBS; pH 7.4).

## Establishment of biomaterials on the metal electrode surface

**Fig. 5:**
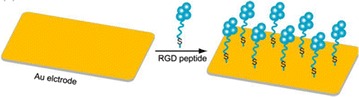
Self-assembly of Cysteine terminated RGD peptide on Au surface. Reprinted with permission from: Ref. [44]. Copyright@American Scientific Publishers

Establishment of biomaterials on the metal electrode surfaces is a challenging task that evolves series of processes such as functionalization with desired functional group for effective immobilization of target functional group of a biomaterials or incorporation of a desired functional group for specific coupling with the functional groups of the target material surface [33, 34]. Material functionalization with large molecules or proteins of whole ECM can be performed with relatively simple Langmuir Blodget (LB) method or self-assembly method [35]. However, modification of artificial surface with small molecules like PLL and RGD peptides requires pre-activation of materials with functional groups. For the PLL immobilization, Au surface was pre activated with 11-MUA that provides negative attraction force for the immobilization of positively charged PLL peptide [4]. On the other hand, for RGD peptide functionalization on Au surface, the RGD peptide itself was modified with an additional cysteine residue suitable for thiol gold coupling where sulfur was covalently linked with gold molecules [10]. Here in, we will discuss detailed about the procedure of RGD peptide immobilization and nanoscale patterning on the Au surface for the fabrication of a highly sensitive cell based chip as shown in Fig. 5.

### Nanoscale RGD peptide thin film preparation

**Fig. 6:**
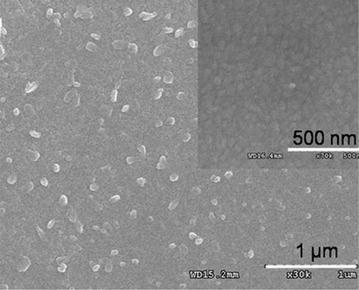
Self-assembly of Cysteine terminated RGD peptide on Au surface. Figure reproduced with permission from: Ref. [2], Copyright (2011) with permission from Elsevier

In vitro nanoscale assembling of molecular building blocks such as nucleic acid, protein and phospholipid, biological organism have been used as versatile tools in nanotechnology. In a recent study, thiolated Au surface was achieved by reacting thiol containing compounds with clean gold surfaces (Fig. 5) [36]. In which sulfhydryl groups of cysteine terminated RGD molecules were covalently bind to the thiolated Au surface and thus allowing the self-assembling of the molecules in two dimensionally over a gold surface [37]. Such RGD peptide modified Au surface is extra useful for establishing mammalian cells for electrochemical measurements because gold conducts electricity and makes for excellent electrical contacts [3, 4]. Therefore, several researches have been conducted with mutagenically modified RGD tripeptides with cysteine residue (an amino acid that contains a thiol group) [33]. RGD molecules were immobilized on the Au surface either as a homogenous thin film or as a definite nanoscale pattern with such engineered molecules using self-assembly or modified self-assembly methods as shown in Fig. 6 [3, 4, 10, 11].

### Formation of Nanopattern RGD peptide layer on Au

Formation of RGD peptide thin film on the Au surface is relatively simple and can be performed using self-assembly method as discusses in the earlier [3, 4]. But, their defined nanoscale pattern on Au surface requires a relatively complex modified self-assembly method [10, 11]. Here in, the detailed steps for the formation of nanoscale RGD pattern using the modified MGSA protocol will be discussed sequentially [10, 11, 18].

**Fig. 7:**
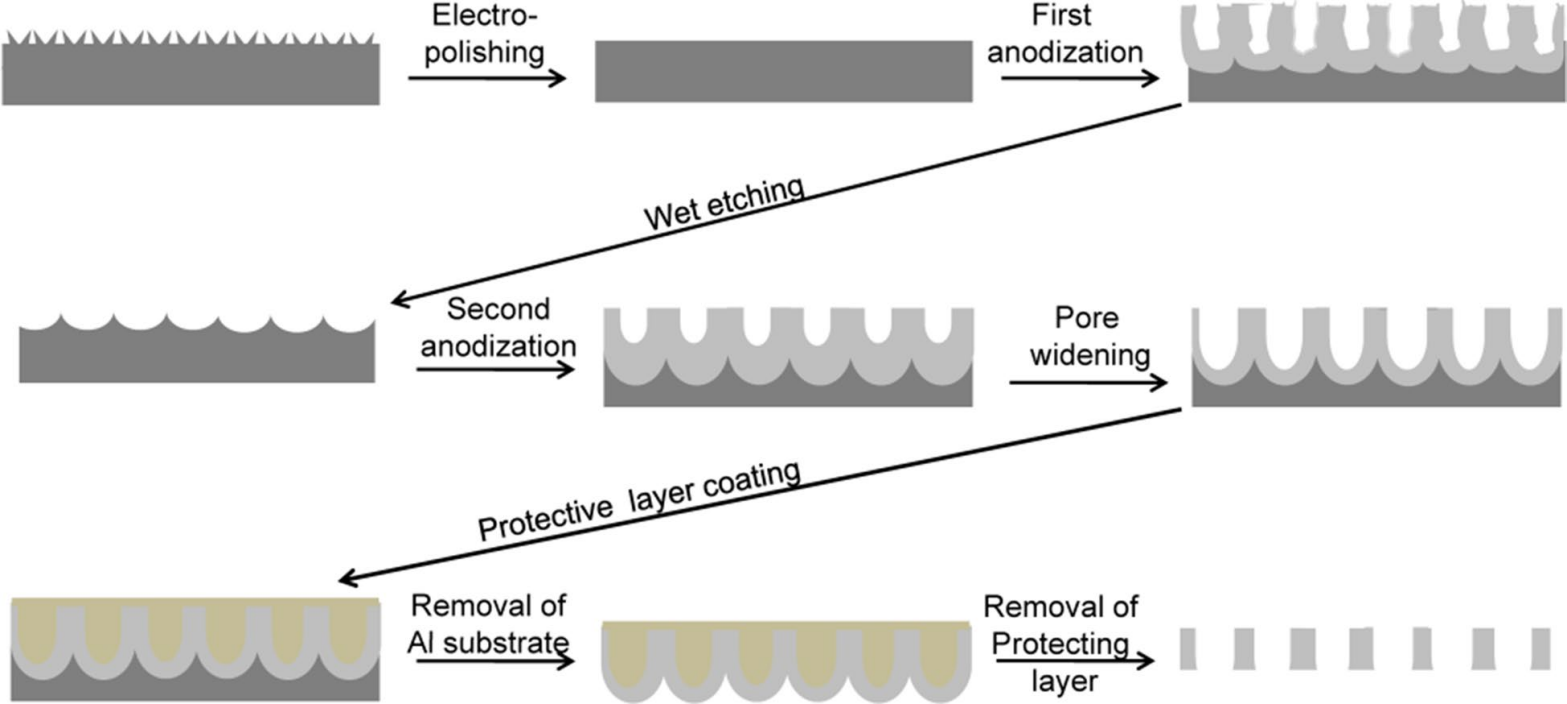
Steps of nanoporous alumina mask synthesis

*Fabrication of Mask and Template Synthesis* Nanoporous alumina template used as a mask for the guided deposition of RGD peptide for achieving the nanopattern as shown in Fig. 7. This porous alumina was fabricated using the two-step anodization process as described elsewhere [10, 11, 38]. Briefly, the surface of the aluminum foil was electro-polished at 20 V in a mixed solution of per chloric acid and ethanol (1:4 in volume) for 60 s. The first anodization was performed by applying a DC voltage of 40 V in 0.3 M oxalic acid solution at 30 °C for 8 h. In order to obtain a well-ordered nanoporous alumina layer, the alumina layer formed during the first anodization process was completely removed by chemical wet etching in a mixture solution of phosphoric acid (0.4 M) and chromic acid (0.2 M) at 650 °C for 4 h. After the removal of the anodic oxide layer, a second anodization process was conducted on the Al substrate under identical conditions to those used for the first anodization [11]. After the second anodization, the surface of the nanoporous alumina was painted with a coating layer consisting of a mixture of nitrocellulose and polyester resin in butyl acetate, ethyl acetate, and isopropyl alcohol. For the preparation of the through-hole alumina masks, the remaining aluminum substrate was removed in a saturated HgCl_2_ solution. Then the alumina barrier layer at the bottom of the cylindrical nanochannel of the alumina layer was etched out in 5 wt% phosphoric acid at 300 °C. To obtain the alumina template with through-holes, the coating layer was dissolved in acetone. The porous alumina was investigated using an atomic force microscope (Nanoscope digital instrument) at a scan rate of 1.00 Hz with phosphorous (n) doped silicon cantilever. The size of the pore was determined by section analysis.

### Formation of 2D/3D RGD peptide nano-patterns

**Fig. 8:**
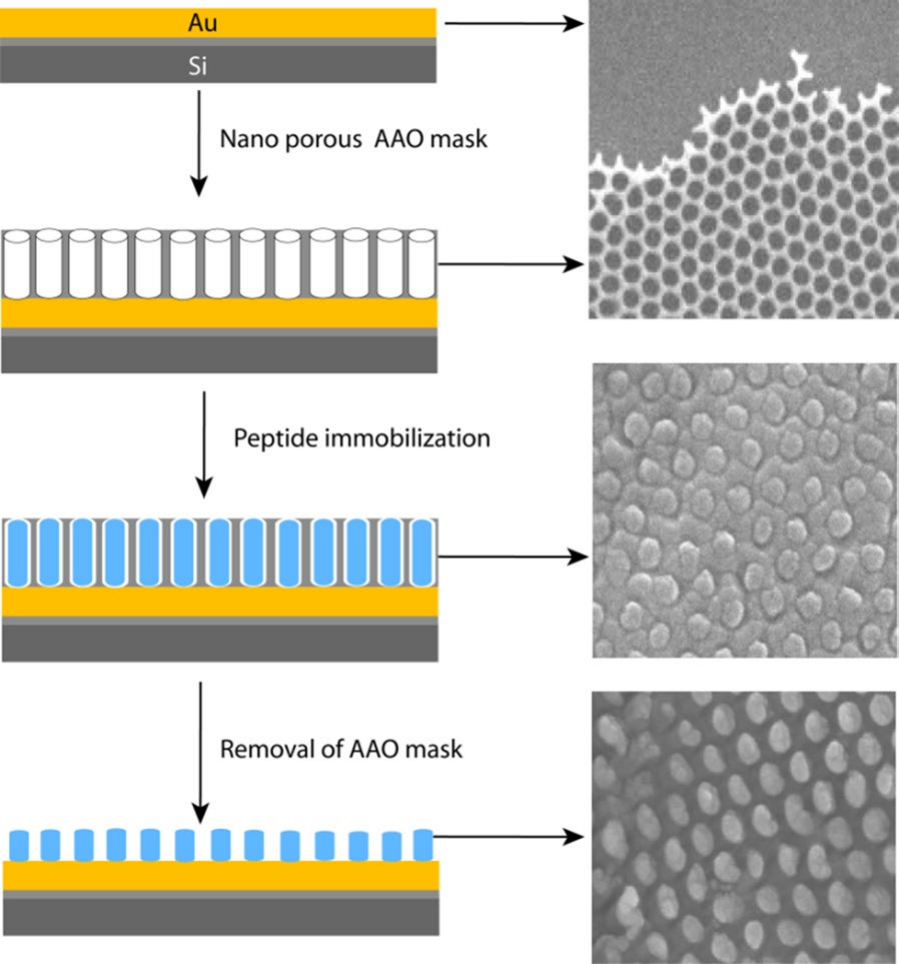
Schematics of mask assisted fabrication of peptide nanodots on Au electrode (*left row*) and SEM images of corresponding steps (*Right row*). Figure reproduced with permission from: Ref. [10], Copyright (2010) with permission from Elsevier

Prior to RGD peptide modification, the Au electrode surface was cleaned thoroughly with piranha solution [38] and dried under nitrogen steam. The nanoporous alumina membrane was placed on the freshly cleaned, smooth Au surface and fixed by adding a drop of acetone. Subsequently, a treatment consisting of 0.01 mg/ml of various peptides diluted with DI water was added separately on the porous alumina membrane and was maintained at 12 h at 40 °C. After deposition, the alumina template was dissolved out using 2 M NaOH followed by rinsing and DIW. In this way, the cysteine-modified RGD peptide was immobilized covalently on gold substrate directly without any organic linker materials. Each sample preparation step was repeated on smooth Au sheets for characterization with scanning electron microscopy (SEM). SEM images were obtained using a field emission scanning electron microscope (Hitachi-S-4300) at an accelerated voltage of 20 kV from Pt/Pd alloy coated samples. The SEM images obtained from various stages of the fabrication processes are shown in the Fig. 8. The SEM images reveals uniformly distributed porous alumina mask was successfully established on the Au surface which was filled with the peptides. Finally, the dot like RGD peptide nanostructures was achieved after removal of the mask.

**Fig. 9:**
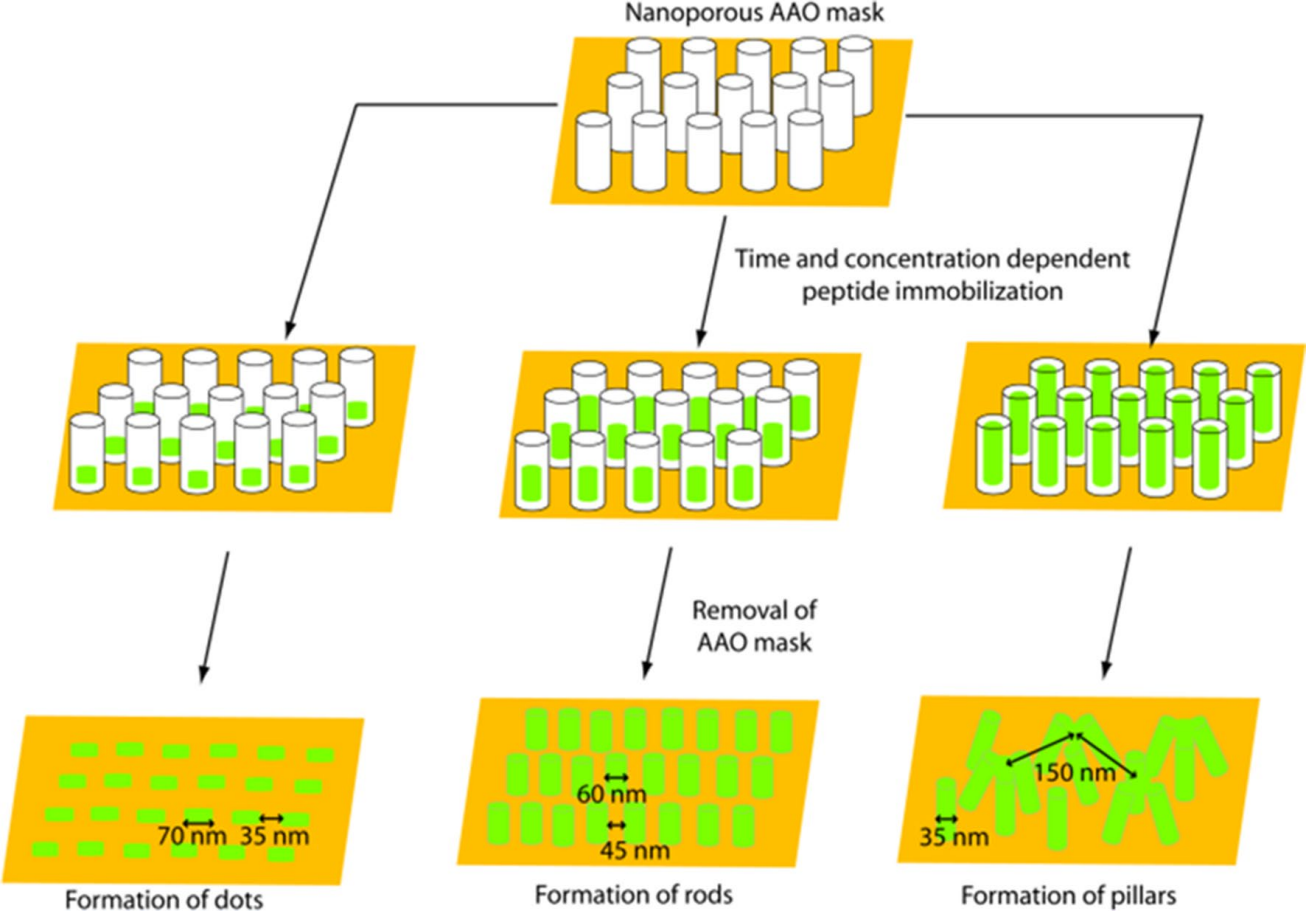
Schematics for the fabrication of various topographic RGD nanopattern. Figure reproduced with permission from: Ref. [11], Copyright (2012) with permission from Elsevier

After achieving the 2D RGD nano-dot, further approaches were undertaken for more precise RGD nano structure-array suitable for in vitro biological activity experiments similar to the in vivo condition [11]. For this nanoporous alumina template with different graded pores (diameters of 74, 63 and 43 nm) were prepared by controlling the pore widening time [11]. These nano-pore diameters showed significant effects on the deposition of peptide and allowed for convenient patterning on Au support. Therefore, using these three different masks, spatially and vertically-controlled 2D-RGD nano-dots, 3D-RGD nano-rods, and 3D-RGD nano-pillars array were achieved when different concentrations of RGD peptide (0.01–0.1 mg/ml) were deposited at 4 °C for 12, 18 and 24 h, respectively as shown in Fig. 9 [11].

**Fig. 10:**
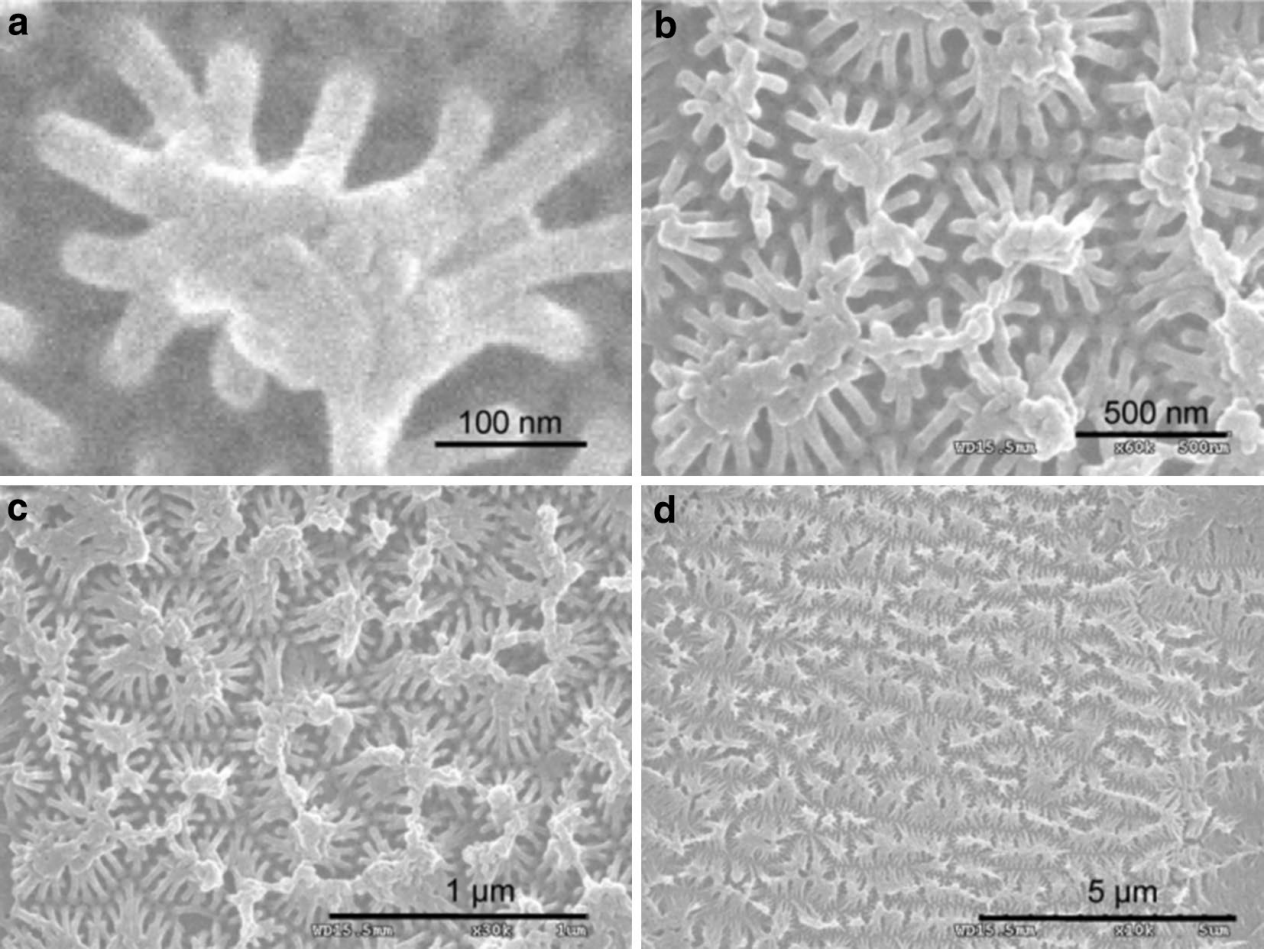
Reproducibility of peptide nano-array synthesis; images obtained from several areas of the fabricated surface at 100 nm (**a**), 500 nm (**b**), 1 µm (**c**), and 5 µm (**d**) *scale bar*. Figure reproduced with permission from: Ref. [11], Copyright (2012) with permission from Elsevier

It was reveals that uniform 2D-RGD nano-dots formed periodic patterns with an average distance of separation of 35 nm. The average diameter of the nano-dots formed on the Au substrate was 70–75 nm. The close-packed hexagonal pore array of the alumina mask played a very important role in determining the ordering of the nanostructures. Therefore, changes in the pore diameter and pore density of the nanoporous masks allowed the diameter and density of the nano-dot array to be modified. It was reported that well-ordered rods are formed due to the reduction of pore diameter and increase in deposition time [39, 40]. Therefore, the experiment was repeated using more precisely controlled pores (43 nm) to achieve further vertical growth of the rod. As a result more vertical growth was observed, resulting in a pillar-like structure with a featured diameter of 35–40 nm and a spatial distance of about 65–70 nm (Fig. 9). Figure 10a shows the 3D-RGD nano-pillar array, where 10–12 pillar heads coalesced to form an integrin receptor site at a distance of around 125-150 nm. Reproducibility of the 3D-RGD nano-pillar arrays is shown in Fig. 10b–d. A large area of the nano-pillar array is shown with different scale bars, 500 nm (Fig. 10b), 1 µm (Fig. 10c), 5 µm (Fig. 10d), and zoomed image of a single scaffold (Fig. 10a) for cellular integrin receptor [14].

## Role of biomaterials on cell adhesion on the metal electrode surface

**Fig. 11:**
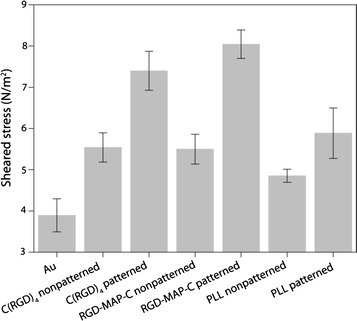
Measurement sheared stress for eruption of PC12 cell immobilized on Au/C(RGD)_4_, Au/RGD-MAP-C, Au/PLL fabricated Au electrode. Figure reproduced with permission from: Ref. [10], Copyright (2010) with permission from Elsevier

Establishment strong link at the cell electrode interfaces is prerequisite for developing an effective cell based chip. Because cell must be firmly attached with an electrode surface to overcome several washing steps require during treatment of analytes as well as during electrochemical measurements. Therefore, several studies have been performed on the selection of biomaterials as well as on the suitable patterning of a biomaterial to ensure farm cell attachment [10, 12, 15, 41, 42]. Our group performs a series of individual research to compare the suitability of Collagen, PLL and RGD peptides for establishing living cells on the metal electrode surface [10]. The results reveal that comparatively stronger cell adhesion ability was achieved from the collagen and RGD peptide modified surface than PLL modified electrode surface (Fig. 11).

The collagen forms thicker layer on the metal surfaces which provide excellent support for cell adhesion, spreading and proliferation, but acts a mechanical beerier for the electron exchange at the cell-electrode interfaces [28, 33]. On the other hand, RGD peptide sequences form homogenous thin layer over the electrode which provides sufficient attachment motif for integrin receptor available on the cell surface [4, 29, 43]. The nanopatterned RGD peptide modified surface provides better cell adhesion ability compared to their homogenous thin film like arrangement [10]. This is because of the spatial arrangement of the RGD peptide specific with the receptor availability of the integrin motif on the cell surface [12, 15]. In addition, the various topographic arrangements of the RGD tripeptide sequences on the artificial surfaces found to affect cellular adhesion, proliferation and differentiation. In our previous research it was reveals that RGD nano pillar like arrangement provides the best possible effect on cell functions compared their RGD nano-rod and dot like arrangements [11]. The nano-rod like arrangement proves better cell immolation ability than their nano dot like arrangement. These materials were proved successful for establishment of rat pheochromocytoma cell (PC-12), Human neuroblastoma cells (SH-SY5Y), Human epithelial carcinoma cells (HeLa), and Human embryonic kidney cells (HEK-293T) on the metal electrode surfaces [3, 4, 10, 11].

**Fig. 12:**
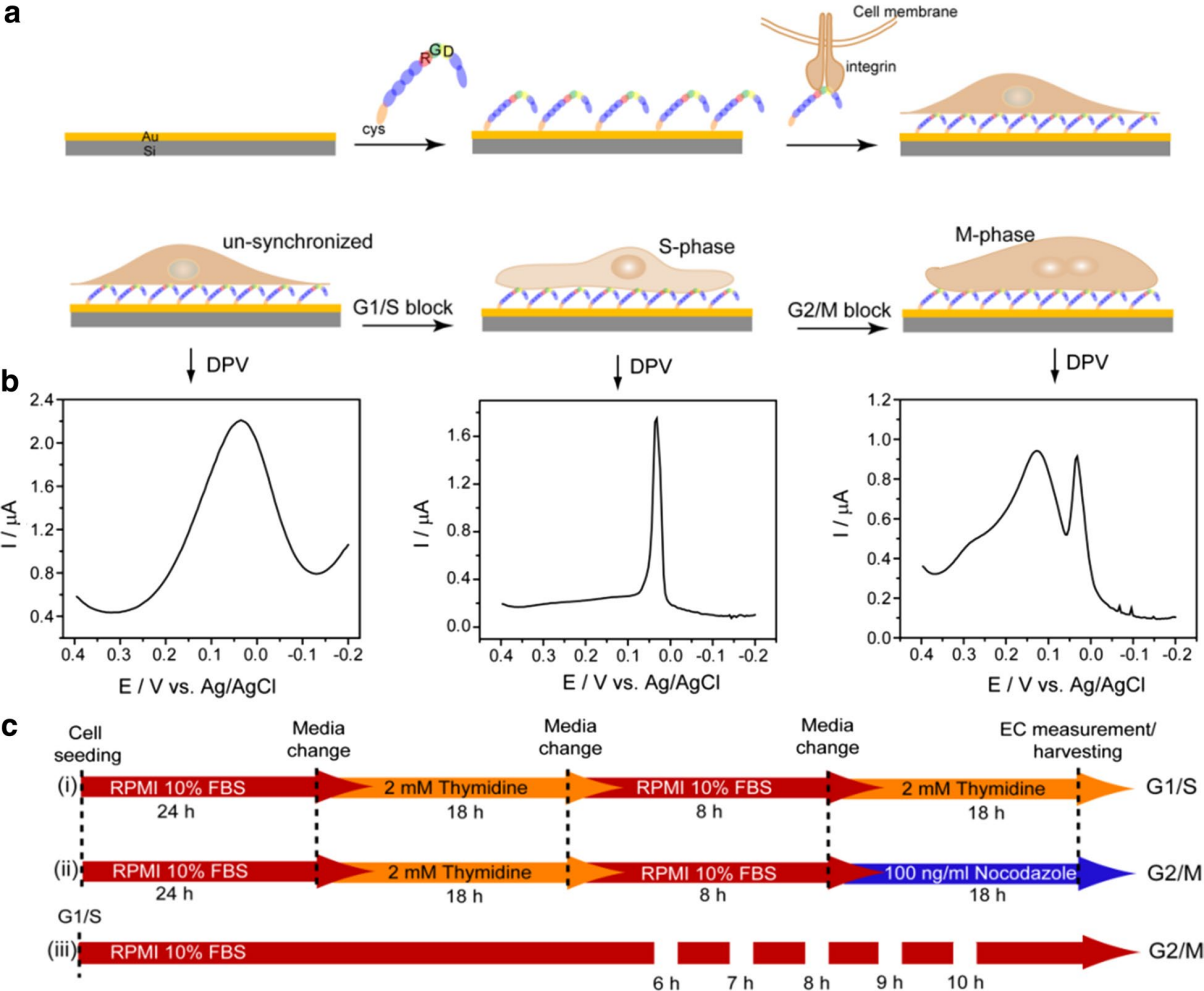
Schematics of experimental setup: **a** fabrication of RGD-MAP-C based-cell chip used throughout the experiments, **b** synchronized G1/S-phase (*middle*), G2/M-phase (*right*), and unsynchronized (*left*) cells with their respective DPV signals (*down arrows* indicate respective signals), and **c** time course of cell treatment for synchronization in G1/S-phase (*i*) and G2/M-phase (*ii*), and gradual progression of G1/S cells towards G2/M-phase following time-dependent release from G1/S block (*iii*). Figure adapted with permission from Ref. [18]. Copyright (2011) American Chemical Society

In a recent study, the RGD nano structured platform was used for the electrochemical determination of cell cycle determination successfully as shown in Fig. 12 [18]. This process required several treatments and withdrawal of treatments for achieving the synchronized at a definite stage (Fig. 12). PC12 cell immobilized on the RGD nanopillar modified electrode surfaces were found to be suitable for such treatment as well as with stand the multiple washing steps that confirm the farm cell electrode attachment. Based on these numerous applications RGD tripeptide sequences were considered as the most suitable material to establish the strong cell electrode interaction and can be used for the development of new generation cell based chip for the onsite monitoring analytes.

## Role of biomaterials on electrochemical measurements

Electrochemical measurement of a cell based chip depends on the conductivity of the electrode material, proper cell electrode interaction and the actual electro-physiologic state of the cell. The electrode materials conductivity is a pre-requisite for any electrochemical devise. Therefore, conductivity of the material must be ensured during designing a chip. Cell-electrode interaction is also an important arena of cell chip based research that has significant impact on the sensitive measurement of a chip. Usually adhesion molecules, proteins and peptides are used for insuring the proper cell electrode interaction of a cell based chip. Several studies have been undertaken to search for a suitable material to maintain proper cell-electrode interaction for increasing cell adhesion as well as for enhancing electron transfer efficiencies [2, 3, 10, 28]. Our previous study reported that large proteins or peptides modified surfaces form a mechanical barrier at the cell electrode interface that impaired electron exchange [10, 28]. Therefore, this review focuses on the engineering of the short adhesion molecules on the electrode surface for increasing cell adhesion without affecting electrode exchange phenomenon [10, 11, 44].

**Fig. 13:**
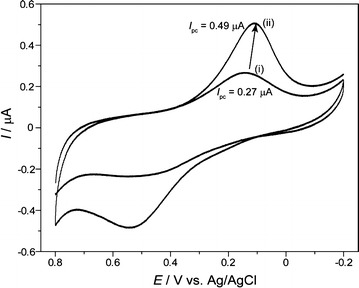
Electrochemical signal of cyclic voltammetry for HEK-293 cells immobilized on bare Au (i) and peptide fabricated Au (ii) surface. Figure reproduced with permission from: Ref. [3], Copyright (2010) with permission from Springer

Engineering of biomaterials on the cell-electrode interface has become an important issue in the recent decade. Engineered ECM materials or its components proteins or peptide has been proved successful for establishing farm cell adhesion as well as their spreading, proliferation and differentiation [12, 13, 15]. However, only few smaller proteins or peptides are found to be suitable for electron transfer between the cell and electrode [2, 3, 10]. Because the whole ECM materials or its larger proteins components like collagen forms a mechanical barrier for electron exchange phenomenon between cell and electrode surfaces. Hence, most of the study recognized a short RGD tripeptide sequences of the cell adhesion motif acts as an effect material for using at cell electrode interfaces [3, 4, 10, 18]. The electrochemical signal was reported to be enhanced when cell immobilized on thin RGD factionalized Au surface (Fig. 13). The actual role of RGD peptide on the enhancement of electrochemical signal of RGD peptide modified electrode yet to be cleared. But their positive effect on cell function has been proved by a number research all over the world [11–13, 15]. Therefore, it is assumed that enhancement of the electrochemical signal might be due to the enhanced spreading and proliferation of cell on the RGD modified electrode.

**Fig. 14:**
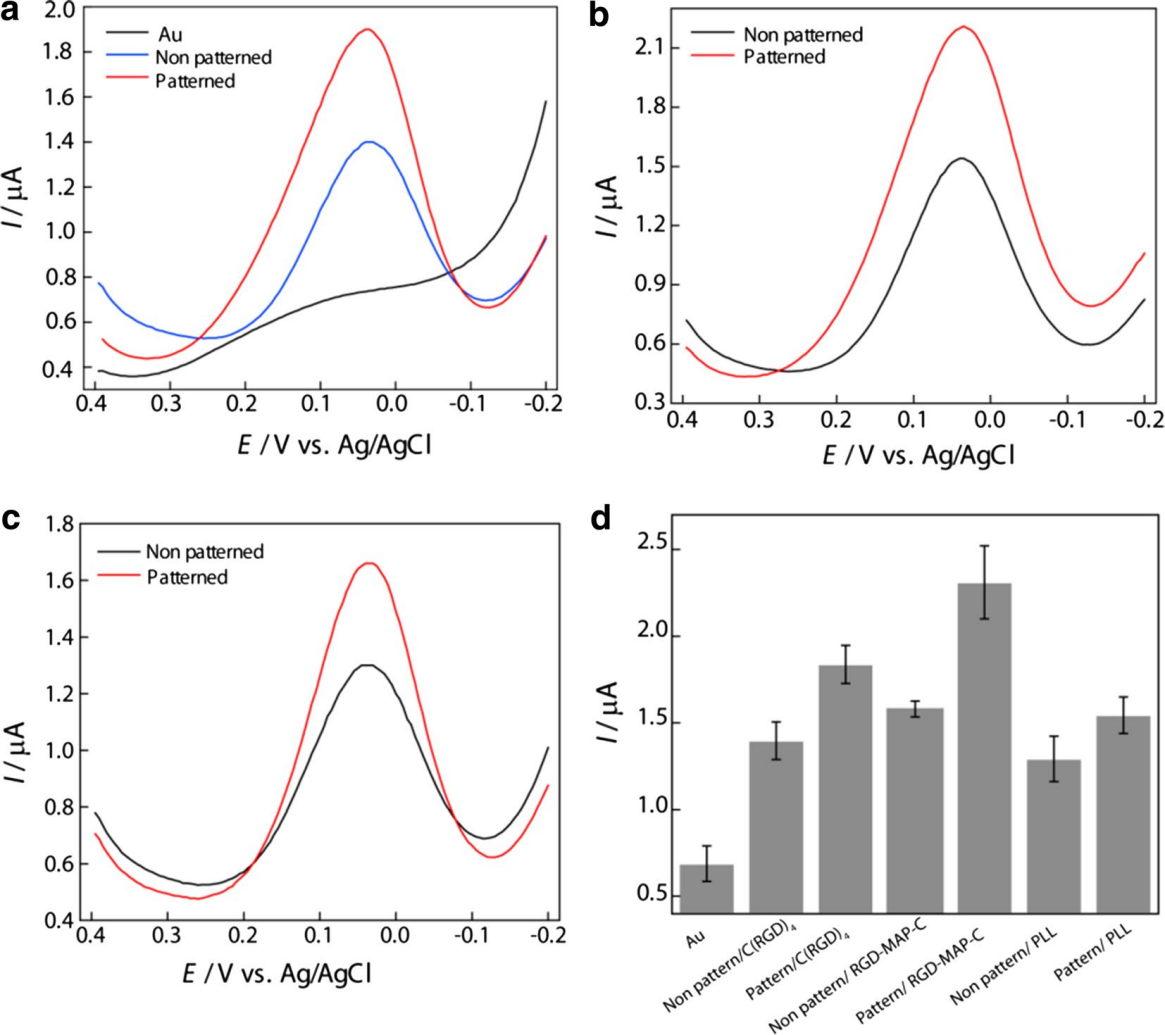
Differential pulse voltammetry of PC12 cell to compare the effects of patterned and non-patterned peptides on Au electrode, **a** C(RGD)_4_, **b** RGD-MAP-C, **c** PLL in PBS (0.01 M pH 7.4). Pulse amplitude and pulse width adopted were 50 mV and 50 ms, respectively. **d** Comparison between DPV peak current from PC12 cells on the different peptide fabricated Au surface. Figure reproduced with permission from: Ref. [10], Copyright (2010) with permission from Elsevier

In addition, the nanoscale pattern of smaller protein or peptide found to pose positive influence on electrochemical measurement. For example, nanoscale patterned PLL and RGD peptides were found to be more suitable for achieving enhanced electrochemical signal compared to their homogenous nanoscale thin film like arrangement (Fig. 13d). In addition to spatial arrangement, the morphological features of the RGD tripeptides i.e. C(RGD)_4_ (linear chain like arrangement) and RGD-Map-C (multi arm arrangement) also influence electrochemical measurements in various ways [10]. More specifically, in an experiment C(RGD)_4_, RGD-Map-C and PLL were immobilized on electrode surface as homogenous thin film or as a predefined nanoscale pattern. The nanoscale patterned surfaces provides higher electrochemical signal than their homogenous thin-film like arrangement (Fig. 14a–c). This feature of peak enhancement is common for all materials used at the cell electrode interfaces because the cellular receptor specific patterned biomaterials forms focal adhesion leaving free space for electron exchange. Therefore, cell grows preferentially on highly-ordered peptide modified electrode surfaces that provide better electrochemical signals [11].

## Conclusions

The whole cell based chip has become a potential tool for electrochemical monitoring or sensing environmental toxicity and in vitro drug effect study. This important tool requires a living cell immobilized conductive surface and a transducer device. The suitability of the cell chip largely depends on cell-electrode interaction force and electron exchange phenomenon between them. The biomaterials having cell adhesion molecules/motif has great impact on the electron exchange mechanism as they are readily employed at the cell electrode interfaces. Therefore, this review focuses on the selection of suitable biomaterials and their engineering on the electrode surfaces to insure farm cell-electrode adhesion as well as to enhance electron exchange between them. The detail discussion reveals that biomaterials like whole ECM materials or its components proteins (laminin, collagen etc.) or small peptides (PLL, RGD etc.) can be used for adhesion and proliferation of cell on metal electrode surfaces. However, most of the large protein when immobilized on the electrode surface they form as thick layer which cause mechanical hindrance of electron transfer between cell and electrode. On the other hand, the small protein or their peptide like RGD, PLL etc. form a homogenous thin film over the electrode which result farm cellular attachment without hampering the electron exchange mechanism. Considering this benefit series of research has been performed on the engineering of the small peptide at the cell electrode interface. The major outcome of such researches include development of protocols for the formation of RGD peptide homogenous thin-film formation and spatially or vertically controlled 2D or 3D nanostructures for enhancing sensitivity of the chip. Most of the research reported that nanostructured RGD tripeptide modified electrode found to be more suitable than their homogenous thin-film like arrangement. Among the fabricated nanostructures RGD nano pillars were found to be more suitable than RGD nano dots. In addition to the spatial and archistructural arrangement, the several polymorphs of synthetic RGD (CRGD_4_, RGD-Map-C) peptides also have influence on the sensitivity of cell based chip. RGD-Map-C was suitably engineered on the electrode surface for the electrochemical monitoring of the cell cycle. Moreover, cell chip with RGD-Map-C nanostructured modified conductive surface was proved to be a suitable platform for onsite electrochemical monitoring of environmental analytes. Therefore, further miniaturization and automation of the chip can improve its application in environmental monitoring or drug effect study.
